# Chronic Obstructive Pulmonary Disease among Patients Visiting the Emergency Department of a Tertiary Care Centre: A Descriptive Cross-sectional Study

**DOI:** 10.31729/jnma.7746

**Published:** 2022-08-31

**Authors:** Kabir Thakali, Dinesh Kumar Lamsal, Sabitra Thapa, Iswor Karki, Ashok Paudel, Manish K.C. Gautam

**Affiliations:** 1 Department of General Practitioners and Emergency Medicine, Nepalese Army Institute of Health Sciences, College of Medicine, Sanobharyang, Kathmandu, Nepal; 2 Department of Family and Emergency Medicine, Civil Service Hospital of Nepal, New Baneshwor, Kathmandu, Nepal; 3 Dhaulagiri Hospital, Baglung Bazar, Baglung, Nepal

**Keywords:** *chronic obstructive pulmonary disease*, *emergency departments*, *prevalence*

## Abstract

**Introduction:**

Chronic obstructive pulmonary disease is characterised by persistent airflow limitation which is usually progressive and is the primary global cause of morbidity and mortality. It is the third leading cause of Years Lived with Disability, the second most common cause of death after ischemic heart disease, and the fourth most common cause of premature death. The aim of this study was to find out the prevalence of chronic obstructive pulmonary disease among patients visiting the emergency department of a tertiary care centre.

**Methods:**

A descriptive cross-sectional study was done in the Department of Emergency Medicine of a tertiary care centre from 4 July 2022 to 11 July 2022. Ethical approval was obtained from the Institutional Review Committee (Reference number: 24). Data from 348 patients were collected from the hospital records. Convenience sampling method was used. Point estimate and 95% Confidence Interval were calculated.

**Results:**

Among 348 patients visiting the emergency department, 23 (6.60%) (6.57-6.63, 95% Confidence Interval) had chronic obstructive pulmonary disease. The mean age of these patients was 73.50±2.76 years.

**Conclusions:**

The prevalence of chronic obstructive pulmonary disease was lower than in the previous studies done in similar settings. The study could provide a general idea of the burden of the disease.

## INTRODUCTION

Chronic obstructive pulmonary disease (COPD) is defined as a preventable and treatable disease characterised by persistent airflow limitation that is usually progressive and associated with an enhanced chronic inflammatory response in the airways and the lung to noxious particles or gases.^1^ COPD is a leading source of chronic morbidity and mortality around the world; many people suffer from it for years before succumbing to it or its complications.

Because of continued exposure to COPD risk factors and population ageing, the global burden of COPD is expected to rise in the coming decades.^2^ As per the study done by the Nepal Health Research Council the prevalence of COPD in Nepal was 11.70% prevalence of COPD in Nepal.^3^ This type of study has not yet been conducted in similar settings to the best of our knowledge.

The aim of this study was to find out the prevalence of COPD among patients visiting the Emergency Department in a tertiary care centre.

## METHODS

A descriptive cross-sectional study was conducted in Civil Service Hospital of Nepal, Kathmandu, Nepal from 4 July 2022 to 11 July 2022. Ethical approval was taken from the Institutional Review Committee of Civil Service Hospital of Nepal (Reference number: 24). The study included all the patients above 18 years of age presenting to the Emergency Department whereas missing and incomplete hospital records were excluded. Convenience sampling method was used. The sample size was calculated by using the following formula:


$$\begin{array}{ll} \text{n} & ={\text{Z}}^{2}\times \frac{\text{p}\times \text{q}}{{\text{e}}^{\text{2}}}\\ & =1.96^{2}\times \frac{0.117\times 0.883}{0.04^{2}}\\ & =249 \end{array}$$

Where,

n= minimum required sample size
Z= 1.96 at 95% Confidence Interval (CI)
p= prevalence taken as 11.7%^3^
q= 1-p
e= margin of error, 4%

The minimum required sample size obtained was 249. A 10% was added to address the missing data after which the minimum required sample size was calculated to be 276. However, a sample size of 348 was taken for the study. The diagnosis of COPD was made by the attending physicians based on the clinical history, examination findings, and radiological and epidemiological manifestations. COPD severity was determined on the basis of global initiatives for chronic obstructive lung disease (GOLD) guidelines.^4^

The necessary data were collected from the hospital records after receiving consent from the emergency department of the same hospital. Data regarding the diagnosis, age, gender, presenting complaints and the outcome were collected and then entered in Microsoft Excel 2016. Error and inconsistency were verified after checking the source document and statistical analysis was done using Microsoft Excel 2016. Point estimate and 95% CI were calculated.

## RESULTS

**Figure 1:**
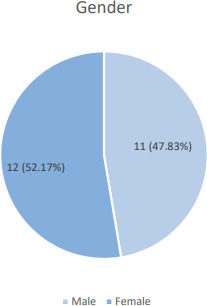
Gender-wise distribution of the patients with COPD (n= 23).

Among 348 patients visiting the emergency department, 23 (6.60%) (6.57-6.63, 95% CI) had chronic obstructive pulmonary disease. (Figure 1).

**Figure 2:**
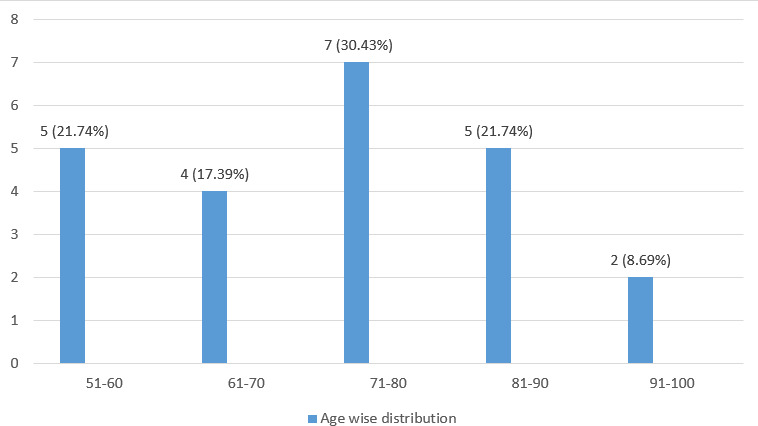
Age-wise distribution of the patients with COPD (n= 23).

The mean age of the patients was 73.50±2.76 years. A total of 7 (30.43%) patients, between the age group of 71-80 have COPD (Figure 2).

The most common presenting complaint of the patients with COPD was shortness of breath, which was seen in 16 (69.57%) followed by cough in 11 (47.83%) patients (Table 1).

**Table 1:** Distribution of presenting complaints among COPD patients (n = 23).

Presenting complaints	n (%)
Shortness of breath	16 (69.57)
Cough	11 (47.83)
Fever	5 (21.74)
Others	7 (30.43)

**Figure 3:**
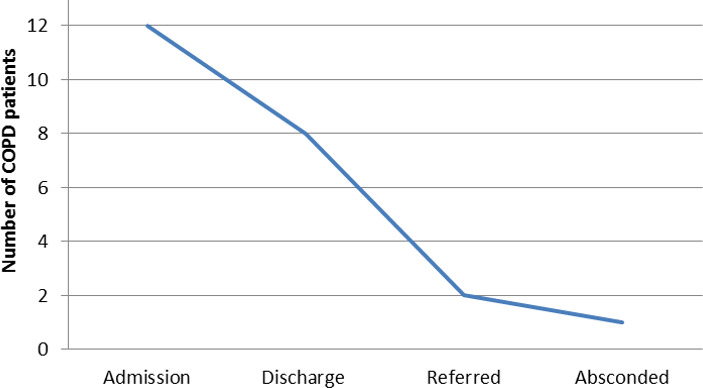
Outcome of the patients with COPD (n= 23).

Out of these 23 patients, 12 (52.17%) were admitted, 8 (34.78%) were discharged, 2 (8.69%) were referred and 1 (4.34%) was absconded (Figure 3).

## DISCUSSION

In this study, the prevalence of COPD among patients in the emergency department was 6.60%. The prevalence reported in this study is lower than the study done by the Nepal Health Research Council which had reported an 11.70% prevalence of COPD in Nepal.^3^ Likewise, the identified prevalence is also lower than the estimate from the Region of the Americas (14.53%) and SouthEast Asia Region/Western Pacific Region (8.80%).^5^ The differences in prevalence might be due to different settings and approaches to studies. Our present study selected patients from the emergency departments with ages more than 18 years over 1 week, whereas NHRC had done a nationwide population-based study over 2 years.^3^ The lower prevalence as compared to the national prevalence might be due to the seasonal distribution of disease exacerbation and timing of the study which can be assumed from the previous study which has shown a decrease in the disease distribution towards the third quarter of each fiscal year.^6^

The majority of the patients were in the 71-80 age group with the mean age of the patient being 73.5±2.76 years which was in contrast to the study conducted among patients admitted in the tertiary hospital of Nepal which showed the majority in 60-70 years with mean age (68.19±10.36).^7^ The majority of the patients with COPD were females (52.2%) which was similar to the systemic review and meta-analysis done on the prevalence and risk factors of COPD in Nepal.^8^ But it is in contrast with the global picture.^9^ The increase in the prevalence of female patients with COPD has also been reported in the study conducted in another tertiary hospital in Nepal.^6^ One large meta-analysis has also highlighted the increasing prevalence of COPD in the female population.^10^ However, another study on gender-specific estimates of COPD prevalence has observed the highest prevalence of males in the South-East Asian region (11.34%).^11^ The gender-based bias has been observed for the diagnosis of COPD in study.^12^ Still, the increased prevalence of COPD in this centre can be attributed to the increased accessibility of health care facilities to women, affordable health services and more female patients visiting the government hospital in Nepal. Nevertheless, more studies should be conducted so as to determine the exact prevalence of COPD in females and to identify possible associated gender-based risk factors. This finding will raise awareness of COPD as a serious problem affecting women's health and make it easier to take into consideration while seeking alternative diagnoses in women.

The majority of the patients with COPD have complaints of shortness of breath 16 (69.56%). Another study conducted in tertiary hospitals in Nepal has shown a higher prevalence of dyspnoea in patients visiting the emergency department (8.90%).^13^ However, our study showed that the prevalence of shortness of breath in an emergency department is 4.60%. This highlights the importance of accurate diagnosis of COPD so as to exclude other possible and frequently encountered alternative diagnoses. Other studies have shown that the breathlessness of COPD is often confused with asthma^14,15^ and association of breathlessness with negative psychological impact.^15^ Thus, breathlessness in COPD should be taken with great importance while managing and diagnosing COPD which has a role in both morbidity and mortality and accurate disease diagnosis as well.

There were certain limitations in the findings of this study. The small sample size and the single centre included in the study could limit the generalizability of the findings. Also, because of the nature of the study design, the association between the variables and outcomes with causality could not be established. Larger studies with a bigger sample size are recommended. The cross-sectional design of the study did not include follow-up of the patients.

## CONCLUSIONS

The prevalence of COPD was lower than the previous studies done in similar settings. However, the seasonal variation and majority of cases attending the outpatient department might have affected the results.The finding has also supported the evidence of rising COPD prevalence in the female population. The health care professionals attending the emergency department should be trained and educated regarding the prompt identification and management of COPD-related cases. The tertiary centre should make proper arrangements for the management of those cases. Further study is warranted to understand the variations in COPD-related emergency visits and hospital admissions.
